# Glutathione S-Transferase Enzymes in Plant-Pathogen Interactions

**DOI:** 10.3389/fpls.2018.01836

**Published:** 2018-12-21

**Authors:** Gábor Gullner, Tamas Komives, Lóránt Király, Peter Schröder

**Affiliations:** ^1^Plant Protection Institute, Centre for Agricultural Research, Hungarian Academy of Sciences, Budapest, Hungary; ^2^Research Unit for Comparative Microbiome Analyses, Department of Environmental Sciences, Helmholtz Zentrum München, German Research Center for Environmental Health (GmbH), Neuherberg, Germany

**Keywords:** bacterium, fungus, glutathione S-transferase, oxidative stress, plant pathogen, salicylic acid, virus, WRKY

## Abstract

Plant glutathione S-transferases (GSTs) are ubiquitous and multifunctional enzymes encoded by large gene families. A characteristic feature of *GST* genes is their high inducibility by a wide range of stress conditions including biotic stress. Early studies on the role of GSTs in plant biotic stress showed that certain *GST* genes are specifically up-regulated by microbial infections. Later numerous transcriptome-wide investigations proved that distinct groups of *GST*s are markedly induced in the early phase of bacterial, fungal and viral infections. Proteomic investigations also confirmed the accumulation of multiple GST proteins in infected plants. Furthermore, functional studies revealed that overexpression or silencing of specific *GSTs* can markedly modify disease symptoms and also pathogen multiplication rates. However, very limited information is available about the exact metabolic functions of disease-induced GST isoenzymes and about their endogenous substrates. The already recognized roles of GSTs are the detoxification of toxic substances by their conjugation with glutathione, the attenuation of oxidative stress and the participation in hormone transport. Some GSTs display glutathione peroxidase activity and these GSTs can detoxify toxic lipid hydroperoxides that accumulate during infections. GSTs can also possess ligandin functions and participate in the intracellular transport of auxins. Notably, the expression of multiple *GSTs* is massively activated by salicylic acid and some GST enzymes were demonstrated to be receptor proteins of salicylic acid. Furthermore, induction of *GST* genes or elevated GST activities have often been observed in plants treated with beneficial microbes (bacteria and fungi) that induce a systemic resistance response (ISR) to subsequent pathogen infections. Further research is needed to reveal the exact metabolic functions of GST isoenzymes in infected plants and to understand their contribution to disease resistance.

## Introduction

**Figure 1:**
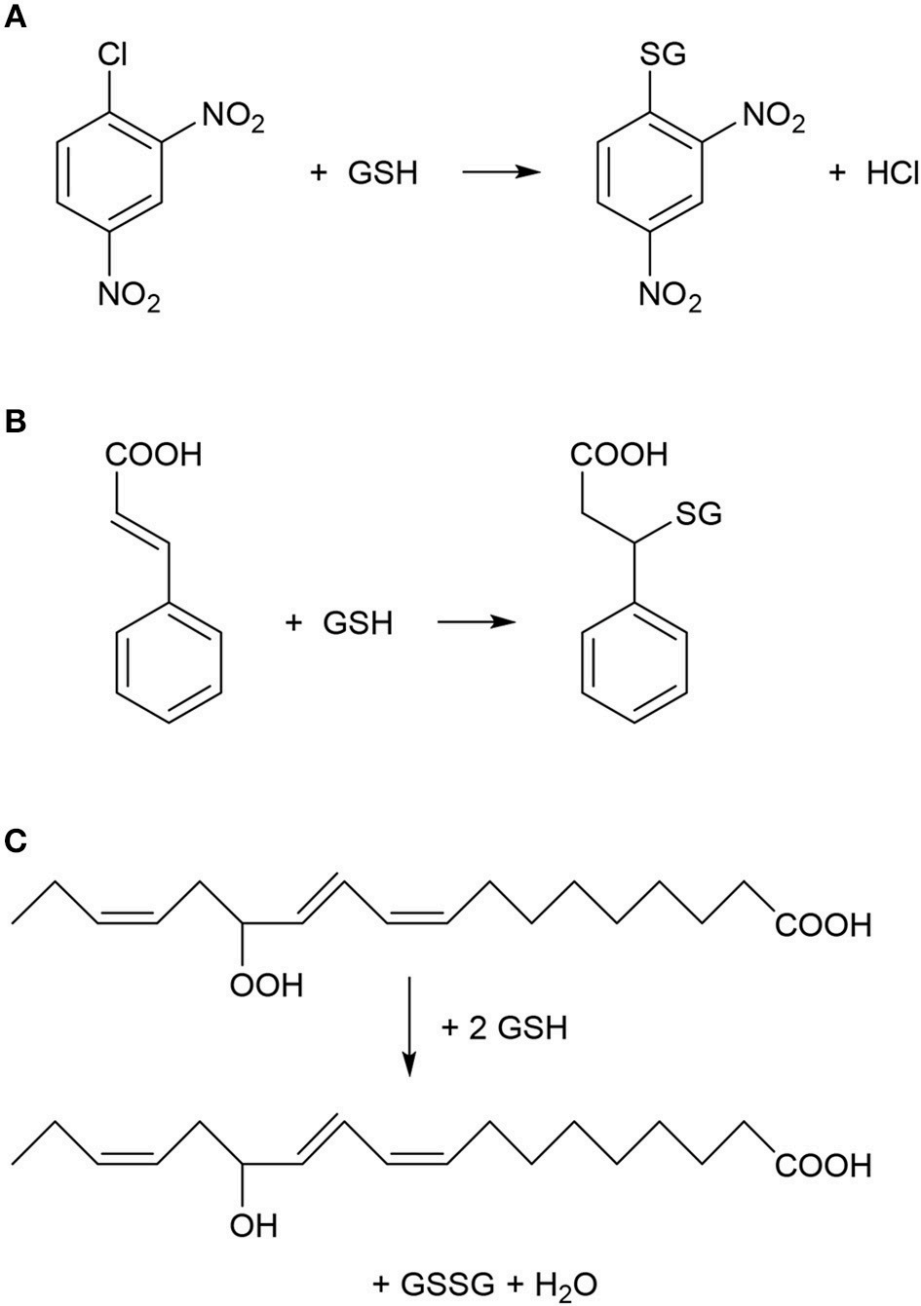
Typical chemical reactions catalyzed by plant glutathione S-transferase (GST) enzymes. **(A)** Nucleophilic substitution reaction between 1-chloro-2,4-dinitrobenzene (CDNB) and reduced glutathione (GSH). CDNB has been extensively used as a xenobiotic model substrate for GST activity determination (Habig et al., 1974). **(B)** Nucleophilic addition reaction between cinnamic acid and GSH (Edwards and Dixon, 1991). **(C)** Reduction (detoxification) of fatty acid hydroperoxides to corresponding hydroxy derivatives by the peroxidase activity of GST as described by Bartling et al. (1993). The substrate 13(S)-hydroperoxy-9,11,15-octadecatrienoic acid was found to accumulate during membrane-damaging lipid peroxidation in infected plants (Wagner et al., 2002).

The first reports about a plant glutathione S-transferase enzyme (GST, EC 2.5.1.18) appeared in 1970, when it was revealed that a GST catalyzed the detoxification of the herbicide atrazine by its conjugation to the endogenous tripeptide glutathione (GSH, γ-L-glutamyl-L-cysteinyl-glycine) in sorghum and maize plants (Frear and Swanson, 1970; Lamoureux et al., 1970). These initial results sparked an intensive GST research, which focused on the detoxification of various herbicides and other toxic xenobiotic compounds in plants (Lamoureux and Rusness, 1989; Timmerman, 1989; Dixon et al., 1998; Schröder et al., 2007). GSTs were shown to catalyze the conjugation between various xenobiotics with electrophilic centers (Figure 1) and the nucleophilic GSH, thus tagging the xenobiotic for vacuolar sequestration (Martinoia et al., 1993). The resulting GSH or homoglutathione (γ-L-glutamyl-L-cysteinyl-β-alanine) conjugates were usually much less toxic and more water-soluble than the original xenobiotics (Brown and Neighbors, 1987; Dixon et al., 1998). Importantly, it was revealed that multiple GST enzymes possess also glutathione peroxidase activities, thus these GSTs can participate in antioxidative defense (Figure 1) (Bartling et al., 1993; Wagner et al., 2002; Dixon et al., 2009). Plant GSTs are mostly cytosolic, and they can represent up to 2% of soluble proteins (Pascal and Scalla, 1999). Several GSTs were shown to be auxin-inducible and to bind auxins as non-substrate ligands (ligandin function) as well as to participate in auxin transport (Bilang and Sturm, 1995; Droog et al., 1995). Furthermore, it was revealed that GSTs play a role during the normal metabolism of plant secondary products like anthocyanins (Marrs, 1996). Nevertheless, in contrast to the vast knowledge collected about the detoxification function of GSTs, the understanding of their role in endogenous plant processes and about their metabolic substrates had been still far from complete (Marrs, 1996; Edwards et al., 2000; Dixon and Edwards, 2009; Dixon et al., 2010).

With the advent of “omics” technologies it was soon recognized that GST enzymes are encoded by large gene families in plants (McGonigle et al., 2000; Wagner et al., 2002). The genome of the model plant *Arabidopsis thaliana* harbors 54 *GST* genes, which were grouped into seven distinct classes in plants (Dixon et al., 2002, 2009). The well-studied large phi (GSTF) and tau (GSTU) classes are specific to plants whereas the small zeta (GSTZ) and theta (GSTT) classes exist also in animal tissues (Dixon et al., 1998, 2002). Less information is available about the three outlying minor GST classes including lambda GSTs (GSTL), dehydroascorbate reductases (DHARs), and tetrachlorohydroquinone dehalogenase (Dixon et al., 2002, 2009). In many cases *GST* genes displayed high inducibility by diverse abiotic and biotic stimuli (DeRidder et al., 2002; Wagner et al., 2002; Sappl et al., 2009; Dixon et al., 2011; Csiszár et al., 2014). Notably, multiple *GST* genes were shown to be strongly inducible by the key defense hormone salicylic acid (SA) (Fodor et al., 1997; Sappl et al., 2004, 2009). More recently GSTF2, GSTF8, GSTF10 and GSTF11 were identified as SA-binding receptor proteins in *A. thaliana*, but the biological relevance of SA binding to these GSTFs still remains to be explored (Tian et al., 2012).

Plants use a sophisticated surveillance system to recognize signals of microbial pathogens (Jones and Dangl, 2006). The investigation of the mechanisms whereby pathogens elicit defense responses in plant cells is of key importance to the understanding of plant disease resistance. Resistance is determined by the timely recognition of the pathogen and by the rapid deployment of efficient plant defense reactions (incompatible plant-pathogen interaction). Late and weak host defense reactions, however, result in susceptibility and disease (compatible interactions). Recognition of pathogens in resistant plant genotypes activates several consecutive downstream signaling cascades. Signals are transmitted to the nucleus leading to the rapid and extensive reprogramming of gene expression patterns in host plant cells (Chisholm et al., 2006; Boller and He, 2009). Resistance is often associated with the accumulation of reactive oxygen species (ROS) and programmed cell death at the sites of infection (hypersensitive response, HR) (Barna et al., 2012; O'Brien et al., 2012). In this regard, GSTs that also possess glutathione peroxidase activities may play a crucial role in plant antioxidative defense by limiting the excessive spread of HR-associated cell death (Levine et al., 1994; Wagner et al., 2002). It should be noted, however, that resistance and HR (programmed cell death) do not necessarily correlate (Bendahmane et al., 1999; Künstler et al., 2016).

Intriguingly, the marked accumulation of multiple GST transcripts and proteins as well as elevated total GST enzyme activities have often been observed in various plant-pathogen interactions. In addition, functional studies of individual GSTs proved in several cases that these enzymes can positively contribute to antimicrobial resistance in host plants by mostly unknown mechanisms (Dixon et al., 2009, 2010; Sappl et al., 2009; Liao et al., 2014; Wahibah et al., 2018). A clearly recognized function of GSTs is their participation in antioxidative reactions together with the pivotal cellular antioxidant GSH in order to eliminate ROS and lipid hydroperoxides that accumulate in infected tissues (Figure 1) (Wagner et al., 2002). Furthermore GSH, which is the most important non-protein thiol compound in plants, plays important roles in both signaling and defense reactions in infected plants (Datta et al., 2015; Gullner et al., 2017; Hernández et al., 2017).

Since the beginning of plant GST research a massive amount of information has been gathered on the role of GSTs in various plant-pathogen interactions (reviewed earlier by Gullner and Komives, 2001, 2006). The present review is an attempt to summarize the most important findings on GSTs in fungus-, bacterium- and virus-infected plants with a special attention to the possible functions of GSTs in disease resistance.

## GSTs in Plant-fungus INTERACTIONS

Numerous pathogenic fungi that infect plants are biotrophic, since they require live plant cells and tissues for host invasion. On the other hand, necrotrophic fungi obtain nutrients by killing infected tissues of the plant host. Hemibiotrophs are a third group of plant pathogenic fungi characterized by an early biotrophic phase of pathogenesis later converting into a necrotrophic lifestyle (Barna et al., 2012; Spanu and Panstruga, 2017). In this section, firstly the contribution of GSTs to interactions of plants with biotrophic fungi are discussed in a historical context, followed by the description of physiological roles of GSTs in infections caused by hemibiotrophic and necrotrophic fungi.

### Biotrophic Fungi

A pioneering paper reported in 1991 the first evidence on the participation of a specific GST in the interaction between wheat and the biotrophic fungal pathogen powdery mildew. Winter wheat (*Triticum aestivum*) infected with the non-adapted pathogen (i.e., eliciting nonhost resistance in wheat) barley powdery mildew (*Blumeria graminis* f. sp. *hordei*, formerly *Erysiphe graminis* f. sp. *hordei*) developed local, induced resistance against a second infection with wheat powdery mildew (*B. graminis* f. sp. *tritici*). The onset of this resistance correlated with the activation of defense genes including a 20-fold increase in the transcript abundance of a *GST* gene (*GstA1*) in wheat leaves infected with *B. graminis* f. sp. *hordei* (Dudler et al., 1991). The *GstA1* gene, which encodes a 29 kD GST protein (GST29) was specifically inducible by fungal infections and exogenous GSH, but not by various xenobiotics that typically induce GST activity (paraquat, atrazine, alachlor, metolachlor) (Mauch and Dudler, 1993). The transcript abundance of *GstA1* increased dramatically within 2 h after infection with barley or wheat powdery mildew. However, in the incompatible and compatible interactions the level and time course of *GstA1*expression were similar. The accumulation of *GstA1* mRNA was also induced following inoculation with another fungal pathogen, *Puccinia recondita* f. sp. *tritici*. It was supposed that GST29 likely prevents plant cell disruption and death caused by highly toxic radicals that accumulate during infection, localizing thereby the host response during HR (Mauch and Dudler, 1993). Some years later total GST enzyme activity was measured in three barley cultivars inoculated with barley powdery mildew. A marked (3.6-fold) elevation of GST activity was found in infected leaves of a very susceptible barley cultivar, while the GST activity increased only to a much lesser extent in moderately susceptible and resistant cultivars. These results imply that GSTs are not associated with the resistance of barley against powdery mildew (El-Zahaby et al., 1995). The above findings were later confirmed by a report, in which a powdery mildew-susceptible barley line (*Hordeum vulgare* cv. Ingrid) and related near-isogenic lines expressing different resistance genes (*Mla12, Mlg*, or *mlo5*) were inoculated with *B. graminis* f. sp. *hordei* race A6. Activities of GST and some antioxidative enzymes were markedly induced 5–7 days after inoculation in susceptible barley leaves. Less significant pathogen-induced enzyme activity changes were detected in *Mla*-type resistant plants that showed HR-type cell death following inoculation, and, to an even lesser extent, in *Mlg* and *mlo* lines with no visible symptoms accompanying the incompatible interaction (Harrach et al., 2008). In addition, infection of *A. thaliana* plants with the biotrophic powdery mildew fungus *Erysiphe orontii* led to the up-regulation of pathogenesis-related (*PR*) genes and a *GST*. No differences were observed in the expression of this *GST* between wild-type *A. thaliana* and its mutants displaying enhanced disease susceptibility (Reuber et al., 1998).

In contrast to the above results, in some cases GSTs were shown to contribute to resistance against powdery mildew. In a gene chip study of wheat–wheat powdery mildew interactions, the up-regulation of ROS-eliminating genes was observed including those encoding DHAR, glutaredoxin, peroxidase, and GST enzymes. The comparison of resistant and susceptible wheat biotypes revealed that the *GSTF5* gene was more strongly induced in the incompatible interaction than in the compatible one (Wang et al., 2012). Furthermore, responses of tomato against the biotrophic fungal pathogen tomato powdery mildew (*Oidium neolycopersici*) were compared between incompatible and compatible interactions. A GST was more rapidly up-regulated in resistant wild tomato plants (*Solanum habrochiates*) harboring the *Ol-1* resistance gene than in susceptible plants. Virus-induced gene silencing was used to knock-down the expression of this *GST* gene in resistant plants, and the *GST*-silenced plants showed a susceptible phenotype after inoculation with *O. neolycopersici*. The resistance against *O. neolycopersici* was associated with HR. These results indicated that a *GST* was required for resistance against *O. neolycopersici* in tomato (Pei et al., 2011).

The expression of *GSTs* was functionally characterized in *A. thaliana* plants in response to treatment with herbicides, phytohormones, oxidative stress and inoculation with virulent and avirulent strains of the obligate biotrophic downy mildew oomycete *Hyaloperonospora parasitica* (formerly *Peronospora parasitica*). The abundance of AtGSTF6 transcripts was up-regulated by all treatments while AtGSTF2, AtGSTF8, AtGSTU19, and AtGSTZ1 showed a selective individual spectrum of inducibility to the different stresses suggesting that regulation of gene expression is controlled by multiple mechanisms (Wagner et al., 2002). Transcriptome profiling using whole genome Affymetrix microarrays of soybean (*Glycine max*) plants exposed to the rust pathogen *Phakopsora pachyrhizi* identified 112 differentially expressed genes, including a markedly induced *GST* (Panthee et al., 2007). A similar transcriptome profiling was conducted in resistant and susceptible genotypes of *Glycine tomentella* following *P. pachyrhizi* infection. Genes encoding stress and defense response-related proteins including GSTs were up-regulated consistently in infected plants (Soria-Guerra et al., 2010).

A proteomics approach was used to compare compatible and incompatible interactions of wheat and the biotrophic yellow rust pathogen *Puccinia striiformis* f. sp. *tritici*. A matrix-assisted laser desorption/ionization time-of-flight mass spectrometry (MALDI-TOF MS) assay revealed several proteins with antioxidant functions including a GST that were differentially expressed between compatible and incompatible interactions, indicating the differential accumulation of ROS in infected tissues (Li et al., 2011).

### Hemibiotrophic Fungi

The important role of GSTs in antifungal plant resistance was demonstrated also in hemibiotrophic plant-fungus interactions. The late blight oomycete *Phytophthora infestans* was shown to activate a *GST* gene (*prp1-1*) in potato. The levels of PRP1-1 mRNA as well as protein rapidly increased in potato leaves after fungal infection. Photoaffinity labeling of this GST with tritiated 5-azido-indole-3-acetic acid suggested that the phytohormone indole-3-acetic acid (IAA) serves as a regulator or substrate of the enzyme (Hahn and Strittmatter, 1994). In *Nicotiana benthamiana* infected by the fungi *Colletotrichum destructivum* and *C. orbiculare*, expression of two genes encoding GSTs (*NbGSTU1* and *NbGSTU3*) was markedly induced. Remarkably, the resistance toward *C. orbiculare* was highly suppressed in *N. benthamiana* when the transcription of *NbGSTU1* was blocked by gene silencing: 67% more colonization and 130% more lesions caused by *C. orbiculare* was observed as compared to control plants. These results unequivocally demonstrated that one GST gene/isoenzyme in *N. benthamiana* certainly has an important role in resistance to hemibiotrophic fungal pathogens (Dean et al., 2005).

In contrast to the above results, a *GST* gene cloned from roots of tobacco (*Nicotiana tabacum*) infected by the hemibiotrophic oomycete *Phytophthora parasitica* var*. nicotianae* was demonstrated to be required for disease susceptibility. The resistance of tobacco markedly increased against the fungus in plants that were GST-silenced. These observations show that individual GST genes/enzymes may suppress plant resistance in the initial biotrophic phase of the infection, possibly by providing a high antioxidative capacity favorable to the fungus (Hernández et al., 2009).

A cDNA library enriched for defense response mRNAs was constructed by suppression subtractive hybridization of sorghum tissues infected with *Colletotrichum sublineolum*, which causes the devastating anthracnose disease. A *GST* was induced in the resistant cultivar but its expression was hardly detectable in susceptible plants, suggesting that this GST may play a significant role in anthracnose resistance (Li et al., 2013). Furthermore, GSTs catalyzed the conjugation of cinnamic acid with GSH in suspension cultured cells of legume species (Figure 1). The activity of this bean GST was increased 2- to 3-fold by exposing plant cells to an elicitor prepared from cell walls of the fungal bean pathogen *Colletotrichum lindemuthianum* (Edwards and Dixon, 1991). In *A. thaliana*, GSH and indole glucosinolates were shown to exert key functions in the immune system. A tau class GST (GSTU13) was identified as an indispensable component of an immune pathway producing defensive indole glucosinolates. The lack of functional GSTU13 resulted in enhanced disease susceptibility toward several fungal pathogens including *Erysiphe pisi, Colletotrichum gloeosporioides*, and *Plectosphaerella cucumerina* (Pislewska-Bednarek et al., 2018).

A tau GST gene, *LrGSTU5*, isolated from *Lilium regale* was found to be markedly inducible by signaling agents like SA and ethylene as well as after inoculation with the soilborne, hemibiotrophic fungal pathogen *Fusarium oxysporum*. In order to verify *LrGSTU5* gene function, a constitutive plant expression vector of *LrGSTU5* was transferred into tobacco. Defense-related genes encoding osmotin, PR-1b, chitinase, and superoxide dismutase (SOD) enzymes were up-regulated in the transgenic lines as compared to wild-type plants. In addition, three important antioxidant enzymes, GST, SOD, and ascorbate peroxidase (APX), displayed significantly higher activities in transgenic lines after inoculation with *F. oxysporum*. Notably, the antifungal resistance of transgenic *LrGSTU5-*overexpressing tobacco lines against *F. oxysporum* infection was markedly increased (Han et al., 2016).

An important aspect of GSH metabolism in fungus-infected plants is the detoxification of fungal toxins (mycotoxins) by GSTs of host plants. Trichothecenes are an important group of mycotoxins that are produced by several phytopathogenic fungi, including the hemibiotrophic *Fusarium graminearum*. Treatment of barley spikes with the type B trichothecene deoxynivalenol (DON) led to the marked up-regulation of gene transcripts encoding e.g., GSTs. The formation of DON-GSH conjugates was also observed. These results showed that GSH-conjugation catalyzed by GSTs may reduce the impact of trichothecenes (Gardiner et al., 2010). Furthermore, a highly up-regulated *GST* gene was identified by a microarray approach in peanut in response to *Aspergillus parasiticus*, which is a saprophytic mold fungus producing carcinogenic aflatoxins (Luo et al., 2005).

### Necrotrophic Fungi

Necrotrophic fungal pathogens destroy host plant tissues usually by toxins and feed on the remains of dead cells. ROS play a central role during plant–necrotrophic fungus interactions by stimulating the plant's defense responses. To overcome ROS-induced damage, both the host and pathogen developed antioxidant systems to quench excess ROS (Barna et al., 2012). A typical necrotrophic pathogen is *Botrytis cinerea*, causing the gray mold disease of plants (Veloso and van Kan, 2018). A proteomic study showed that *B. cinerea* infection led to the accumulation of catalase 3 and multiple GSTs in *A. thaliana*, demonstrating the importance of an antioxidant system in defense against the fungus, which is known to cause oxidative stress in infected host tissues (Mulema et al., 2011). In addition, a reprogramming of carbohydrate and lipid metabolism was observed in grape (*Vitis vinifera*) berries infected with *B. cinerea* that resulted in an increased biosynthesis of secondary metabolites involved in plant defense. Genes encoding WRKY transcription factors, PR-proteins, a phenylalanine ammonia-lyase (PAL) and a GST were up-regulated in infected berries (Agudelo-Romero et al., 2015). Grapevine (*Vitis*) species may resist fungal infections by accumulating secondary metabolites like stilbenoid phytoalexins (trans-resveratrols). A tau class GST (GSTU-2) was identified in *V. vinifera* cell cultures and shown to be involved in extracellular transport of trans-resveratrols: grapevine cell cultures overexpressing GSTU-2 accumulated trans-resveratrols in the extracellular medium even without any elicitation of plant defenses or pathogen infection (Martínez-Márquez et al., 2017). However, in *Vitis flexuosa* different GTSs may play diverse roles in pathogen defense, since only one out of five characterized GST genes was induced, while expression of the other GSTs was down-regulated following infection by the necrotrophic fungi *B. cinerea* and *Elsinoe ampelina* (Ahn et al., 2016).

**Figure 2:**
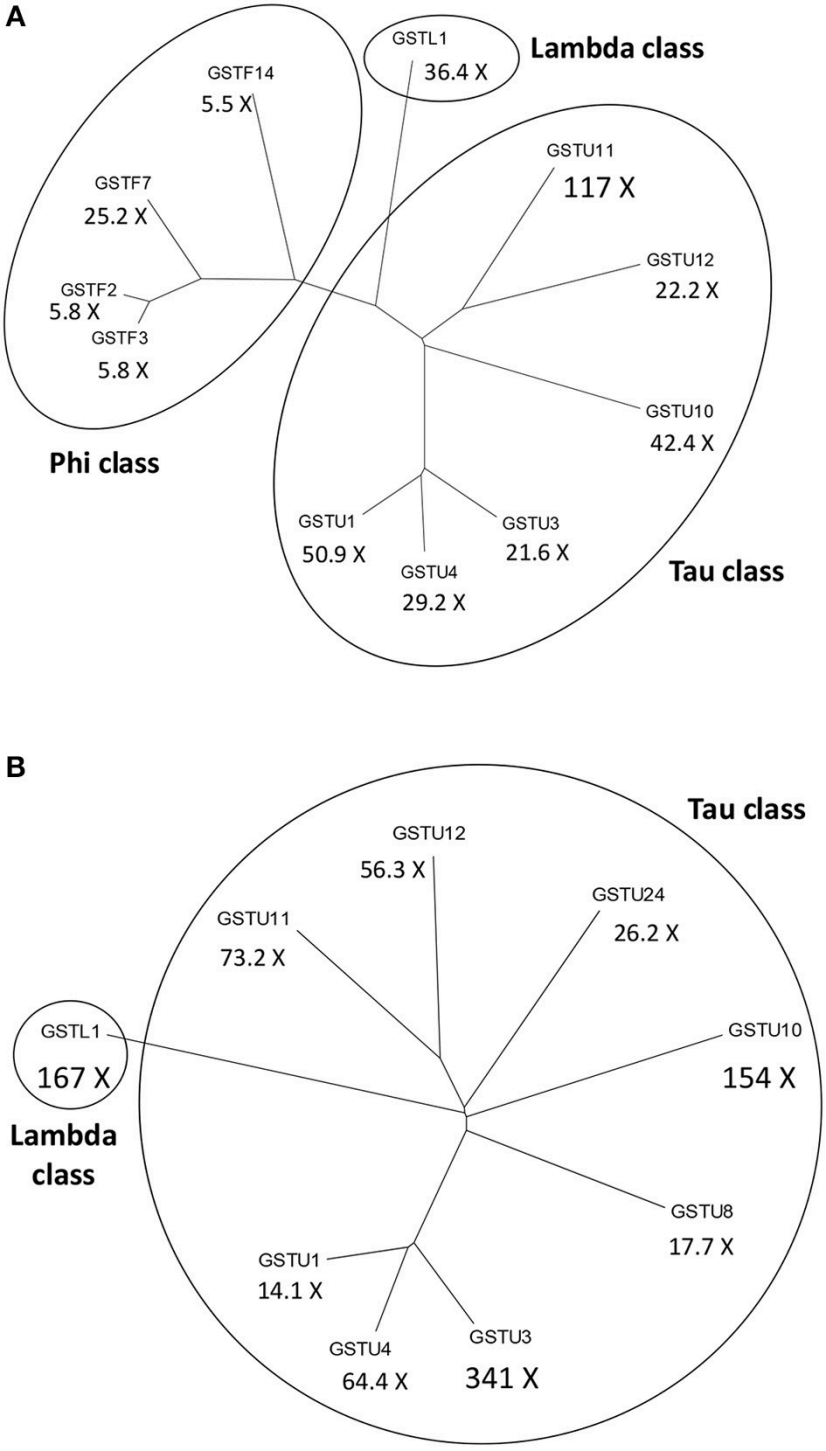
Dendrograms of the most significantly activated *Arabidopsis thaliana* glutathione S-transferase (*GST*) genes following a fungal or a bacterial infection. Below the abbreviated gene names the magnitudes of gene inductions are shown (X =-fold). **(A)** More than 5-fold up-regulated *GSTs* at 48 h following infection of *A. thaliana* with the necrotrophic fungal pathogen *Alternaria brassicicola*. **(B)** More than 10-fold up-regulated *GSTs* at 12 h following infection of *A. thaliana* with the *Pseudomonas syringae* pv. *tomato* DC3000 strain carrying the *avrRpt2* effector gene (incompatible interaction). The expression data obtained by De Vos et al. (2005) were collected from the NCBI Gene Expression Omnibus database.

In leaf tissue of *A. thaliana* inoculated with the necrotrophic fungus *Alternaria brassicicola*, a microarray analysis revealed a significant increase in the abundance of 168 mRNAs. Activation of genes encoding antioxidant enzymes such as catalases and GST1 was detected in the tissue surrounding the initial infection site (Schenk et al., 2000). Changes in the proteome of *A. thaliana* were also studied following *A. brassicicola* infection by two-dimensional gel electrophoresis combined with mass spectrometry. The abundance of several proteins including two GSTs (AtGSTF7 and AtGSTU7) markedly increased (Mukherjee et al., 2010). In a different study, multiple *GSTs* belonging to various GST classes were strongly activated in the leaves of *A. thaliana* following *A. brassicicola* infection (De Vos et al., 2005). Particularly the *GSTU11, GSTU1* and *GSTU10* genes were robustly induced 48 h post-inoculation by *A. brassicicola*. The expression of several *GSTFs* including *GSTF7* and a *GSTL* gene were also markedly up-regulated after the fungal inoculation (Figure 2A).

In a proteomic study, cotyledons of two *B. napus* cultivars resistant and susceptible to the causal agent of stem rot (*Sclerotinia sclerotiorum*) were infected with *S. sclerotiorum* and proteins differentially regulated between the two cultivars identified. Certain enzymes accumulated only in the resistant oilseed rape cultivar following inoculation, such as those related to antioxidative defense including a GST, to ethylene biosynthesis, protein synthesis and protein folding (Garg et al., 2013). Multiple markedly up-regulated *GST* genes were also observed by a microarray approach in partially resistant oilseed rape cultivars following *S. sclerotiorum* infection (Zhao et al., 2007, 2009). To identify resistance genes and PR-genes, five highly resistant and susceptible *B. napus* lines were selected for transcriptome sequencing following inoculation with *S. sclerotiorum*. Twenty-four genes were identified that were differentially expressed in resistant or susceptible genotypes, including a tau class *GST* (*GSTU*) gene cluster (Wei et al., 2016; Seifbarghi et al., 2017).

The soil borne necrotrophic fungus *Verticillium dahliae* causes the very destructive Verticillium wilt disease in a wide range of host plants including cotton plants. A genome-wide association study identified the *GaGSTF9* gene in *V. dahliae*-infected tree cotton (*Gossypium arboreum*) as a positive key regulator of resistance against Verticillium wilt. Silencing of *GaGSTF9* in a resistant *G. arboreum* accession resulted in significantly more fungal colonies after *V. dahliae* infection. Transgenic *A. thaliana* plants overexpressing *GaGSTF9* showed significantly lower SA and H_2_O_2_ levels than wild type plants. Upon *V. dahliae*-infection SA levels massively increased in transgenic plants but H_2_O_2_ accumulation was low as compared to wild type plants, indicating that GST may regulate the content of ROS via catalytic reduction with GSH that affects also the SA content (Gong et al., 2018).

### Plant-Fungus Consortium Interactions

GSTs play an important role also in the esca disease of grapevine. The esca disease is a devastating, but still poorly understood fungal disease of grapevine trunks. Several fungi inhabiting the woody tissues were shown to be causal agents of the esca disease complex (Bertsch et al., 2013). The GSH pool decreased and PR-proteins were induced in leaves of esca-infected grapes before the appearance of visible symptoms. In addition, GST activities in leaves, expression of genes encoding GSTU1 and GSTF2 and GSTU1 and GSTF2 protein abundance were highest at early infection stages but decreased as visible symptoms later appeared. GSTF2 was found in the nucleus and in the cytoplasm, whereas GSTU1 was detected mostly in plastids. The expression of *GSTs* and the ratio of GSSG to total glutathione were suggested as early indicators of the presence of the esca disease in grapevine canes (Valtaud et al., 2009; Magnin-Robert et al., 2017).

### Regulation of GST Genes During Plant-Fungus Interactions

Limited information is available about the regulation of plant *GSTs* during fungal infections. Several aspects of regulation of the *A. thaliana GSTF8* have been revealed and this gene has become a marker commonly used for early stress and defense responses (Thatcher et al., 2015). The response of the promoter of *GSTF8* from *A. thaliana* to infection by the soil-borne necrotrophic fungal pathogen *Rhizoctonia solani* was investigated using a luciferase reporter system. Although the reporter gene was induced in infected roots, the response differed markedly between *R. solani* strains and was not observed with aggressive strains that caused death of the seedlings. The induction was observed also in plants harboring a tetramer of the *ocs* element from the *GSTF8* promoter, suggesting that this element helps to mediate the response (Perl-Treves et al., 2004). Interestingly, antioxidant genes of plants and fungal pathogens including *GSTs* were distinctly regulated during disease development in different *R. solani* pathosystems (Samsatly et al., 2018). A forward genetic screen for *Arabidopsis* mutants with up-regulated *GSTF8* promoter activity was conducted by fusing a *GSTF8* promoter fragment to the luciferase reporter gene. The *esr1-1* (enhanced stress response 1) mutant was identified conferring enhanced resistance to the fungal pathogen *F. oxysporum*. It was found that the *ESR1* gene encodes a KH domain-containing RNA-binding protein. Transcriptome sequencing of *esr1-1* revealed altered expression of several genes involved in responses to biotic and abiotic stresses and hormone signaling pathways (Thatcher et al., 2015). An additional complexity in the regulation of *GSTF8* promoter results from the occurrence of multiple transcription start sites (TSS) in this gene, which gives rise to alternate *GSTF8* transcripts. The most 3′ TSS gives rise to the shorter, major message (GSTF8-S) that is much more stress-responsive than the longer transcript (GSTF8-L) originating from an upstream TSS, which encodes the larger form of the protein. Analysis of the GSTF8-L and GSTF8-S proteins demonstrated that GSTF8-L is solely targeted to plastids, whereas GSTF8-S is cytoplasmic (Thatcher et al., 2007).

WRKY transcription factor proteins have often been associated with the regulation of antimicrobial defense reactions in host plants (Eulgem and Somssich, 2007). Constitutive overexpression of a cotton gene encoding a WRKY transcription factor (*GhWRKY39*) in *N. benthamiana* conferred elevated resistance to bacterial and fungal infections. The transgenic plants exhibited enhanced tolerance against oxidative stress and increased transcription of antioxidant genes including a *GST* (Shi et al., 2014). Overexpression of *WRKY70* led to the marked up-regulation of numerous target genes of WRKY70 including *GSTF7* in *A. thaliana* (Li et al., 2004). Notably, WRKY70 was shown to determine the balance between SA-dependent and jasmonate-dependent defense pathways (Li et al., 2004, 2006). The overexpression of *WRKY70* in transgenic *A. thaliana* plants caused enhanced SA-mediated resistance to the biotrophic *Erysiphe cichoracearum*, but compromised the jasmonate-mediated resistance against the necrotrophic *A. brassicicola*. Conversely, down-regulation of *WRKY70* impaired resistance to *E. cichoracearum* (Li et al., 2006). In rice, WRKY45 is a positive regulator of resistance against the hemibiotrophic rice blast fungus *Magnaporthe grisea*. In the SA signaling pathway WRKY45 acts independently of NH1, a rice ortholog of the *A. thaliana* master regulator NPR1. Two defense-related genes, encoding a GST and a cytochrome P450, were regulated downstream of WRKY45, but were not regulated by NH1, suggesting independence of the WRKY45 and NH1 pathways (Shimono et al., 2007).

**Figure 3:**
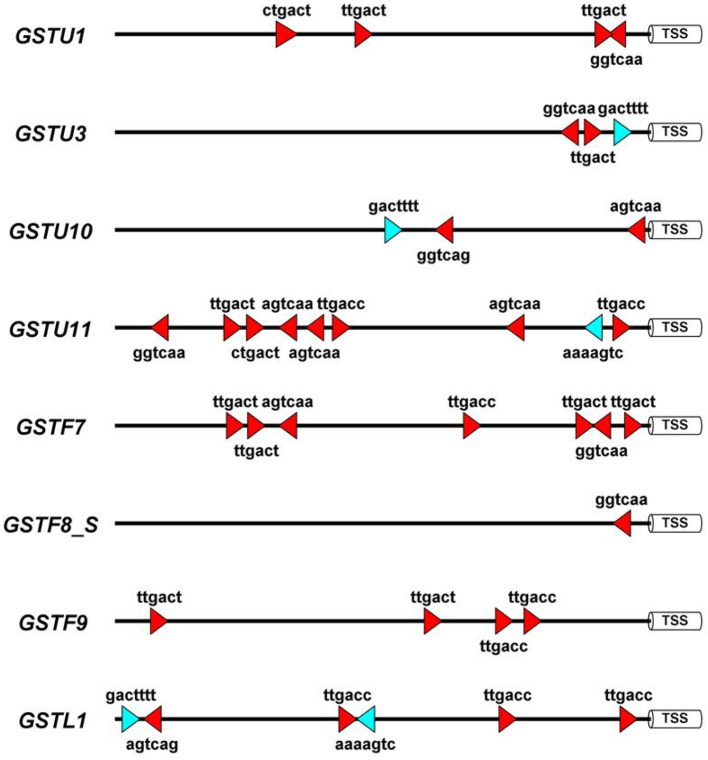
Schematic representation of the disease-related W-box and WT-box *cis*-regulatory elements in the promoter sequences of eight *Arabidopsis thaliana* glutathione S-transferase (*GST*) genes. These sequence motifs are the binding sites of WRKY transcription factor proteins. For *in silico* analyses 1,500 bp DNA segments upstream of the transcription start sites (TSS) were selected from the NCBI GenBank database. In the case of the *GSTF8* gene the promoter of the shorter transcript variant (GSTF8_S) (Thatcher et al., 2007) was analyzed. Symbols: red triangles, W-boxes; blue triangles, WT-boxes. Promoter motifs were found on both DNA strands, which is represented by the orientation of the red and blue symbols. The diagram was prepared by the Illustrator for Biological Sequences (IBS) software (Liu et al., 2015).

To obtain more knowledge on potential roles of WRKYs in *GST* gene regulation we identified the canonical W-box regulatory elements in 1500 bp long promoter segments of eight *A. thaliana GST* genes, which participate in defense reactions (De Vos et al., 2005). These (C/T)TGAC(C/T) motifs have been shown to be pathogen-responsive *cis*-elements that bind WRKY transcription factors (Eulgem and Somssich, 2007). In addition, we also searched for WT-boxes (core sequence GACTTTT), which are the binding sites of WRKY70 in *A. thaliana* (Machens et al., 2014). The number of W-boxes and their distribution patterns highly varied between *GST* promoters (Figure 3). WT-boxes occurred much less frequently in *GST* promoters (1–2 copies) than W-boxes (1–8 copies). Some promoters, like those of *GSTU11* and *GSTF7*, contained an outstandingly large number of W-boxes (8 and 7 copies, respectively) (Figure 3), which was already reported in the case of *GSTF7* (Li et al., 2004). These results suggest that WRKY transcription factors participate in the regulation of *GSTU11* and other *GSTs*, in concert with a large number of other transcription factors and signaling compounds.

### Fungal GSTs

Beside plants, the genomes of plant pathogenic fungi also encode *GST* genes (McGoldrick et al., 2005; Calmes et al., 2015; Sevastos et al., 2017). Fungal GSTs may have a pivotal role in protecting fungi against plant-derived toxic metabolites and ROS accumulating during infection at the host-pathogen interface. Thus, a *GST* gene (*Bcgst1*) was cloned from *B. cinerea*, which was supposed to contribute to the chemical stress tolerance of the fungus. The role of *Bcgst1* in the virulence of *B. cinerea* in tomato was evaluated by constructing gene disruption mutants. Neither of the mutants showed a decrease in virulence, indicating that the *Bcgst1* gene is not essential for virulence on tomato leaves under the conditions tested (Prins et al., 2000). The transcription of a *GST* gene of *A. brassicicola* (*AbGst1*) was significantly enhanced by isothiocyanates, heavy metals and 1-chloro-2,4-dinitrobenzene, but the superoxide-generating menadione and paraquat were inefficient. Isothiocyanates are antimicrobial volatiles produced from glucosinolates by myrosinase enzymes (Bones and Rossiter, 1996). *AbGst1* was up-regulated *in planta* during infection suggesting the potential involvement of this enzyme in isothiocyanate detoxification mechanisms during host plant infection (Sellam et al., 2006, 2007). A more detailed mining of the *A. brassicicola* genome revealed 23 *GST* sequences. Five isothiocyanate-inducible *GSTs* that belong to five different GST classes were more thoroughly investigated. Two GSTs displayed GSH transferase activity with isothiocyanates and peroxidase activity with cumene hydroperoxide substrates. On the other hand, mutants deficient for these two *GSTs* were neither more susceptible to isothiocyanate nor less aggressive than the wild-type parental strain during infection of the host plant *Brassica oleracea*. Three among the five isothiocyanate-inducible GSTs analyzed, were essential for full aggressiveness of *A. brassicicola* on host plants suggesting that GSTs might be essential virulence factors of fungal necrotrophs (Calmes et al., 2015). In addition, multiple GST enzymes identified in the genome of *S. sclerotiorum* participate in the detoxification of isothiocyanates and toxic volatiles from *Brassica* species. This detoxification capacity may allow *S. sclerotiorum* to parasitize tissues of *Brassica* species despite the production of toxic metabolites (Rahmanpour et al., 2009). Also, a *GSTT* gene termed *PiGSTT1* has been cloned from an oomycete pathogen of potato, *P. infestans*. The enzyme PiGSTT1 was shown to be a glutathione peroxidase highly active with organic hydroperoxide substrates like 9(*S*)-hydroperoxy-(10*E*,12*Z*,15*Z*)-octadecatrienoic acid that is synthesized in potato during infection by *P. infestans* (Bryant et al., 2006).

## GSTs in Plant-Bacterium Interactions

Plant-bacterium interactions can lead to three different outcomes: resistance gene (R-gene) mediated resistance, basal resistance and virulence. The *R*-gene mediated, hypersensitive-type resistance (HR, incompatible interaction) is based on a specific interaction, either directly or indirectly, of a bacterial effector gene product with the *R* gene of the host plant. This form of resistance is generally associated with the accumulation of ROS and localized cell death in infected plant tissues. Contrary to the *R*-gene mediated HR-type cell death, recognition in the case of basal resistance is unspecific, as intruders are recognized based on their common molecular patterns. Induction of basal resistance is not associated with visible symptoms, in contrast to the HR-type cell death. An insufficient plant defense results in virulence (compatible interaction) (Truman et al., 2006).

### GSTs in R-gene Mediated Resistance

In HR-type resistance, bacterial infections often cause oxidative stress that leads to the accumulation of ROS including hydrogen peroxide (Baker and Orlandi, 1995; O'Brien et al., 2012). In infected plants, hydrogen peroxide generated during an oxidative stress has a dual role. It may act as a trigger for localized cell death (HR) but also as a rapid signal for induction of antioxidative defenses. An increase in expression of cellular protectant genes occurs at lower doses of H_2_O_2_ than required for HR, and takes place in healthy cells adjacent to necrotic, HR-type lesions in infected leaves (Levine et al., 1994; O'Brien et al., 2012). The up-regulation of plant *GST* genes as a consequence of bacterium-induced oxidative stress was early recognized. H_2_O_2_-accumulation in cell suspension cultures of soybean was shown to be activated by an avirulent strain of the bacterial pathogen *Pseudomonas syringae* pv. *glycinea*. Accumulation of an mRNA encoding a GST was observed as a consequence of this oxidative burst after bacterial infection. However, infection by a virulent strain of *P. syringae* pv. *glycinea* did not result in GST transcript accumulation (Levine et al., 1994). Pretreatment with a tyrosine kinase inhibitor or with a serine/threonine kinase inhibitor inhibited both the oxidative burst and the induction of *GST* in the incompatible interaction (Rajasekhar et al., 1999). The up-regulation of a *GST* gene was observed also in *A. thaliana* inoculated with an avirulent strain of *P. syringae* pv. *maculicola* (Greenberg et al., 1994). Following these early observations, the up-regulation of plant *GST* genes has often been used as an indicator of oxidative stress and HR in plant-bacterium interactions (Alvarez et al., 1998; Desikan et al., 1998; Maleck et al., 2000). However, oxidative stress can occur also in compatible plant-bacterium interactions. The role of GST was also investigated in pear and tobacco infected with the causal agent of fire blight, *Erwinia amylovora*. The bacterium caused GST induction and a sustained oxidative stress in leaves of both pear and tobacco (in compatible and incompatible interactions, respectively). The unexpected fact that *E. amylovora* generates oxidative stress even in compatible plant-pathogen interactions could be linked to its functional *hrp* gene cluster. As suggested by the authors, *E. amylovora* may utilize the production of ROS as a tool to provoke host cell death for a more successful invasion of plant tissues (Venisse et al., 2001).

Bacterial speck disease caused by *P. syringae* pv. *tomato* is one of the most devastating diseases of tomato. The antioxidative ascorbate-GSH cycle was studied in two tomato cultivars infected with *P. syringae* pv. *tomato*. GSH levels, GSH redox ratio and glutathione peroxidase activities were decreased, while the accumulation of GSSG was increased in an inoculated cultivar susceptible to the bacterium. By contrast, in a resistant cultivar the GSH pool homeostasis was maintained throughout the bacterial attack. Moreover, in the resistant interaction a significantly higher constitutive and pathogen-induced GST activity was observed. This research demonstrated the significance of GSH pool homeostasis and GST induction in resistance to *P. syringae* pv. *tomato* (Kuzniak and Sklodowska, 2004). In a more recent study, the expression of selected defense-response genes was investigated in heirloom tomatoes challenged with *P. syringae* pv. *tomato*. Transcript levels of defense genes including *PR-1a*, peroxidase and a *GST* were up-regulated in two resistant cultivars. On the other hand, transcripts from these genes were down-regulated in two susceptible cultivars (compatible interaction). The induction of defense response occurred in the early infection phase at 3 days post-inoculation and it was consistent with lower levels of disease severity in resistant cultivars (Veluchamy and Panthee, 2015). The pepper gene *CaBPR1*, which encodes basic PR1, was strongly induced by ethephon, wounding, and virus infection. Overexpressing *CaBPR1* in tobacco conferred increased tolerance to the oomycete pathogen *Phytophthora nicotianae*, and the bacterial pathogens *Ralstonia solanacearum* and *P. syringae* pv. *tabaci*. The *CaBPR1* transgene increased the expression of the *PR-Q* and *GST* genes (Sarowar et al., 2005).

Microarray expression profiling of the incompatible interaction between *A. thaliana* and *P. syringae* pv. *tomato* DC3000 (*Pst* DC3000) carrying the *avrRpt2* effector (avirulence) gene markedly contributed to the elucidation of plant defense responses in bacterium-infected plants. Thus, data of De Vos et al. (2005) deposited in the GEO database showed that infection with *Pst* DC3000 carrying *avrRpt2* very strongly induced the expression of several *GSTs* in leaves of *A. thaliana* at 12 h post-inoculation, particularly those of *GSTU3, GSTL1, GSTU10*, and *GSTU11* (Figure 2B). Another microarray assay compared early gene expression responses in *A. thaliana* to exogenous SA treatment and to a *Pst* DC3000 strain harboring the effector gene *AvrRpm1*. The presence of this effector gene results in an incompatible plant-bacterium interaction (resistance). Several hundreds of early SA-inducible genes were identified including two *GSTs*. The induction of *GSTU7* and *GSTF8* by SA was independent of the master regulator *NPR1* gene. Examination of the expression patterns for selected early SA-induced genes indicated that their activation by SA required the TGA2/5/6 subclass of transcription factors. These genes were also activated by *Pst* DC3000 *AvrRpm1*, suggesting that they might also play a role in defense against bacteria (Blanco et al., 2009).

*Ralstonia solanacearum* is an important plant pathogenic, soil-borne bacterium, which causes the widespread bacterial wilt disease (Peeters et al., 2013). Northern blot analysis was used to compare expression of defense-related genes in two ecotypes of *A. thaliana* resistant and susceptible to *R. solanacearum* following pathogen inoculation, revealing a significant accumulation of transcripts encoding PR-1, Cu, Zn SOD, and a GST1. In the susceptible ecotype the induction of these defense-related genes was clearly delayed as compared to the resistant one (Ho and Yang, 1999). More recently, a PCR-based suppression subtractive hybridization was carried out to compare defense gene activations between ginger (*Zingiber officinale*) and mango ginger (*Curcuma amada*) leaves following *R. solanacearum* infection. *C. amada* is a potential donor for bacterial wilt resistance to the susceptible *Z. officinale*. Three transcripts were discriminative: the expression of genes encoding a leucine-rich protein, a xyloglucan transglycosylase and a GST was much higher in the resistant species (*C. amada*) than in the susceptible species (*Z. officinale*) at every time point studied (Prasath et al., 2013).

Bacterial leaf blight disease caused by *Xanthomonas oryzae* pv. *oryzae* (Xoo) gives rise to devastating crop losses in rice. The expression of a constitutively active tobacco mitogen-activated protein kinase kinase (NtMEK2^DD^) in transgenic rice plants resulted in HR-like cell death preceded by the activation of endogenous rice 48-kDa MBP kinase, which was also activated by Xoo. The expression of *NtMEK2*^*DD*^ induced the generation of hydrogen peroxide and up-regulated the expression of defense-related genes including *PR-*genes, peroxidases and *GSTs* including *GSTTU4* and *GSTTU12* (Jeong et al., 2008). A transgenic rice cultivar overexpressing the pattern recognition receptor-like kinase Xa21 was used for comprehensive metabolomic and transcriptomic profiling to compare incompatible and compatible rice-Xoo interactions. The rice Xa21 protein confers broad-spectrum resistance against Xoo. Many differential changes occurred in the Xa21-mediated response to Xoo strains. Acetophenone, xanthophylls, fatty acids, alkaloids, GSH, carbohydrate, and lipid biosynthetic pathways were affected. In addition, significant transcriptional induction of several *PR* genes as well as differential changes in multiple GST transcripts were observed (Sana et al., 2010). The accumulation of 16 rice proteins associated with leaf blight was studied by Western blot analysis in various rice-Xoo interactions. The comparison of their accumulation patterns in resistance, susceptible, and mock responses revealed a marked GST accumulation during resistance responses pointing to the role of GST as a positive regulator of resistance (Bai et al., 2012).

External factors, such as light have a strong influence on plant defense reactions and disease resistance. Interaction of *A. thaliana* with an avirulent strain of *P. syringae* pv. *maculicola* in the dark resulted in increased apoplastic bacterial growth and therefore reduced local resistance as compared to infection in light. The extent of oxidative burst, as estimated by induction of a *GST* gene, was not weakened by the absence of light (Zeier et al., 2004). The pathogen-induced expression of *GST1* proved to be higher and faster in younger leaves, whereas the induction of the *PR-1* gene was largely independent of leaf age. Despite these differences in inducible defense, bacterial growth as a measure of disease resistance proved to be similar in inoculated younger and older leaves (Zeier, 2005). Furthermore, diurnal changes were observed in the resistance of tomato against *Pst* DC3000, with the greatest susceptibility before midnight. Nightly red light treatment significantly enhanced the resistance and this effect correlated with increased SA accumulation, defense-related gene transcription and reduced redox homeostasis. Genes involved in redox homeostasis including those encoding GSTs as well as WRKY transcription factors were differentially induced by red light in response to pathogen challenge (Yang et al., 2015).

### GSTs in Basal Resistance

To analyze the early events of basal resistance in tobacco a subtractive hybridization was carried out between leaves treated with the HR-negative mutant strain *P. syringae* pv. *syringae* 61 hrcC and non-treated control leaves. The HR-negative hrcC mutant is still capable to elicit the unspecific, symptomless basal resistance response. Several representative genes associated with basal resistance were identified including a *GST* gene *(EBR-52)* closely related to the auxin-inducible tobacco gene *par-B*. Gene activation patterns showed early peaks 3–12 h after inoculation, paralell with the development of basal resistance. Infection of tobacco with different types of bacteria revealed that incompatible pathogens, their *hrp* mutants, and non-pathogenic bacteria induce high levels of defense gene expression, including that of the above mentioned *GST* (*EBR-52)*, while virulent pathogens induce only a limited response. Furthermore, *GST* (*EBR-52)* expression seems to be specific to bacterial infections as no activation was detected following viral infections (Szatmári et al., 2006).

### GSTs and Virulent Bacteria

In an early report, the accumulation of a *GST* transcript was observed in *A. thaliana* leaves inoculated with the virulent bacterium *Pst* DC3000. This bacterium produces the phytotoxin coronatine that markedly contributes to disease symptom development (lesion expansion, chlorosis formation). Interestingly, a coronatine-deficient mutant bacterium caused only mild symptoms but consistently induced 2- to 5-times higher *GST* transcript levels than the coronatine-producing wild type strain. These results demonstrated that in early stages of infection coronatine may play a critical role by suppressing activation of defense-related genes including *GSTs* (Mittal and Davis, 1995). The expression of the *Pst* DC3000-inducible *AtGSTF2* and *AtGSTF6* genes was shown to be regulated by combined SA- and ethylene-signaling. However, the jasmonate-insensitive *A. thaliana* mutant *jar1* showed normal induction kinetics for both *GSTs* (Lieberherr et al., 2003).

Proteome alterations in leaves of *A. thaliana* during early host responses to *Pst* DC3000 inoculation were analyzed by two-dimensional gel electrophoresis. Protein changes characteristic of virulence, basal resistance and R-gene mediated resistance were assessed by comparing responses to *Pst* DC3000, a *hrp* mutant of the bacterium and a *Pst* DC3000 strain expressing the effector gene *avrRpm1*, respectively. The abundance of selected transcripts was also analyzed in gene-chip experiments. GSTs and peroxiredoxins consistently showed clear differences in abundance after various infections and time intervals. Bacterial challenges generally induced multiple GSTs, however individual members of the GST family were specifically modified depending upon the virulence of bacterial strains and the outcome of interaction. GSTF8 was the only GST to show specificity for the R-gene response. In addition, pathogen challenge elicited particularly dynamic responses of GSTF8: by 2 h after inoculation the corresponding transcript was already significantly up-regulated and the post-translational protein modifications detected were specific for incompatible interactions (Jones et al., 2004). The *GSTF8* gene was also induced by H_2_O_2_ through the activation of MPK3/MPK6 kinases (Kovtun et al., 2000) the promoter of which contains an *as-1* motif, which is implicated in response to oxidative stress (Garretón et al., 2002).

The *A. thaliana* mutant *cir1* (constitutively induced resistance 1) showed enhanced resistance to *Pst* DC3000. Differential gene expression in wild type and *cir1* plants without pathogen challenge were examined using a microarray biased toward defense-response and signaling genes in order to identify transcripts required for resistance. The induction of genes encoding a sodium inducible calcium binding protein, a protein phosphatase, a PAL and GSTF7 were observed (Naidoo et al., 2007).

### Bacterial GSTs

Bacterial genomes also harbor *GST* genes (Vuilleumier, 1997; Kanai et al., 2006; Travensolo et al., 2008; Fang et al., 2011). Genome sequencing projects were particularly useful for the identification of large numbers of *GSTs* of unknown function in bacterial and yeast genomes (Vuilleumier and Pagni, 2002; Skopelitou et al., 2012a,b). Bacterial *GST* genes are often located within gene clusters, which suggests an important role of GST proteins in metabolic degradation and detoxification pathways (Marsh et al., 2008). Bacterial GSTs are implicated in a variety of distinct processes such as the biodegradation of xenobiotics, protection against chemical and oxidative stresses and antimicrobial drug resistance. In addition to their role in detoxification, bacterial GSTs are also involved in other metabolic processes like the degradation of lignin (Allocati et al., 2009, 2012).

## GSTs in Plant-virus Interactions

Plant viruses are obligate biotrophic pathogens that need living tissues for their multiplication. The interaction of plants with the invading virus can be either incompatible (resistance) or compatible (susceptibility) depending on the rapidity and intensity of defense reactions in host plants. In fact, during incompatible plant-virus interactions, the success of resistance at sites of virus infection may also depend on the speed of the host response. Thus, a rapid, efficient host reaction may result in early elimination of viruses and no obvious disease symptoms (extreme resistance). In contrast, a slightly delayed and less efficient host response allows limited virus replication and movement first resulting in oxidative stress and programmed cell death before conferring a final arrest of virus invasion (HR) (Bendahmane et al., 1999; Hernández et al., 2016).

### GSTs and the Hypersensitive Type Resistance

It has been known for decades that treatment of leaves with antioxidants like GSH decrease the number of HR-type necrotic lesions caused by virus infections but virus levels essentially remain the same (Farkas et al., 1960). A paraquat tolerant (i.e., tolerant to oxidative stress) tobacco biotype (*N. tabacum* cv. Samsun) displayed high levels of GSH following e.g., herbicide exposure and enhanced activities of GST associated with reduced development of HR caused by *Tobacco necrosis virus* (TNV) (Gullner et al., 1991, 1995a; Barna et al., 1993). Accordingly, GSTs, in concert with GSH, may have a pivotal function in controlling HR-type necrotization during plant virus resistance, as initially proposed by Fodor et al. (1997). These authors showed that visible HR following *Tobacco mosaic virus* (TMV) inoculation was preceded by a transient drop in antioxidant enzyme activities, e.g., APX, glutathione reductase (GR) and GST. On the other hand, after HR development antioxidant activities and levels of GSH, increased significantly (Fodor et al., 1997). Furthermore, markedly elevated activities of APX, catalase and GST in a cytokinin-overproducing tobacco line were accompanied with a significantly lower number of HR-lesions and reduced levels of TNV, as compared to wild type controls (Pogány et al., 2004). Elevated expression of tau and theta class *GST* genes (*NtGSTU1* and *NtGSTT2*) is also correlated with HR induced by TMV in tobacco (Király et al., 2012; Juhász and Gullner, 2014). In addition, a further increase in *NtGSTU1* expression at 3 and 6 h after virus inoculation was associated with enhanced HR-type resistance (i.e., significantly less necrotic lesions and reduced TMV-replication) in plants with a sufficient sulfate supply (Király et al., 2012).

Enhanced expression of *GST* genes during HR-type virus resistance has been also observed in several other host-virus combinations. For example, the appearance of macroscopically visible lesions in the *A. thaliana* ecotype C-24 resistant to the yellow strain of *Cucumber mosaic virus* (CMV Y) was coupled to elevated induction of a *GST* gene (Ishihara et al., 2004). In pepper, at least two *GST* genes were among the most highly up-regulated defense-related sequences identified in a line resistant to *Capsicum chlorosis virus* (CaCV) at the time point when lesions were fully developed (Widana Gamage et al., 2016).

Importantly, the above results imply that certain GST isoenzymes are not only antioxidants but also have a role in the establishment and/or signaling of virus resistance. This is supported by several additional studies of different plant-virus interactions. For example, purification of virus-host protein complexes from infected plants coupled to mass spectrometry identified a GST co-purifying with *Rice yellow mottle virus* (RYMV) in a partially resistant rice cultivar but not in a susceptible one (Brizard et al., 2006). In sugar beet displaying a strong, symptomless (not HR-type) resistance to *Beet necrotic yellow vein virus* (BNYVV), the causal agent of rhizomania disease, a GST was identified by tandem MALDI-TOF MS. Although this GST was also present in a near isogenic susceptible line, evaluation of corresponding transcript accumulation revealed that *GST* gene expression was significantly induced only in the BNYVV-resistant line (Larson et al., 2008). Comparing gene expression profiles of two rice cultivars showing asymptomatic resistance and susceptibility to *Rice tungro spherical virus* (RTSV) demonstrated the induction of at least twenty GST genes in both interactions. However, almost all of these *GST* genes were expressed to higher levels in the resistant rice cultivar (Satoh et al., 2013).

The importance of GST enzymatic activity in establishing virus resistance has been demonstrated by comparing three sorghum cultivars in their responses to *Sugarcane mosaic virus* (SCMV). The sorghum cultivar GKC-84 displayed a symptomless resistance response (“immunity”) to the virus, which was associated with a more than 50 % increase in GST activity in the first 3 days after SCMV inoculation, while a susceptible cultivar displayed strongly decreased GST activities (Gullner et al., 1995b). Interestingly, a sorghum cultivar of intermediate susceptibility (cv. Róna-2) that develops an initial HR before systemic SCMV spread displayed GST activities intermediate between those of the susceptible and resistant (“immune”) plants. These results suggested that GST activity may be tightly associated with the strength of the virus resistance response. A marked induction of GST isoenzymes could contribute to a strong and possibly early symptomless type of resistance, while a less increase or a decrease of GST activity may confer only a weak virus resistance that eventually results in susceptibility (Gullner et al., 1995b). Furthermore, in a maize cultivar with symptomless SCMV-resistance, a proteomic analysis revealed a down-regulation of two different GSTs in later phases of virus infection, pointing to a role of GSTs in establishing virus resistance at the early stages of pathogenesis (Wu et al., 2013a,b).

### GSTs and Virus Susceptibility

The role of GSTs in inhibiting oxidative stress should be considered not only during HR, but also during virus susceptibility, i.e., systemic infections. Enhanced expression of defense-related genes like *GSTs* during systemic infections could be also due to the silencing suppressor activity of the infecting virus, as shown for *A. thaliana* susceptible to *Beet severe curly top virus* (BSCTV) (Yang et al., 2013). Several *GST* genes were also induced in a RTSV-susceptible rice cultivar that developed no visible systemic symptoms following virus inoculation (Satoh et al., 2013). Similar results were obtained by Casado-Vela et al. (2006) demonstrating a differential expression of antioxidant enzymes, including at least one GST in TMV-infected but asymptomatic tomato fruits. It is tempting to speculate that in cases of systemic virus infections with no or mild symptoms GSTs might significantly contribute to the absence of large scale oxidative stress. Indeed, in *A. thaliana* susceptible to *Cauliflower mosaic virus* (CaMV), compatible infection resulted in the marked systemic induction of *GST1* concomitantly with increased CaMV titers and development of mosaic symptoms (Love et al., 2005). An analysis of soybean susceptible to viruses that cause yellow mosaic disease (*Mungbean yellow mosaic India virus*, MYMIV and *Mungbean yellow mosaic virus*, MYMV) demonstrated the marked accumulation of a GST protein and its corresponding transcript in systemically infected leaves (Pavan Kumar et al., 2017). Furthermore, GSTU10-10 was identified in soybean specifically induced in response to systemic infection by *Soybean mosaic virus* (SMV). Characterization of the GSTU10-10 isoenzyme revealed that it has an antioxidant catalytic function by acting as a hydroperoxidase and has a very low K_m_ for GSH suggesting that GSTU10-10 is able to perform efficient catalysis under conditions where GSH concentrations are low, e.g., during oxidative stress (Skopelitou et al., 2015). A long term systemic infection of peach by *Apple chlorotic leaf spot virus* (ACLSV), the causal agent of “viruela” disease was investigated focusing on changes in host oxidative stress parameters and antioxidant capacity (García-Ibarra et al., 2011). Overall, data showed that systemic infection by ACLSV did not produce any visible symptoms or membrane damage in leaves (i.e., no changes in lipid peroxidation), while antioxidant defenses increased, including GST. Plant defense responses were analyzed in potato (cv. Desiree) leaves systemically infected with *Potato virus X* (PVX). The appearance of mild-yellowish, mosaic symptoms was associated with a dramatic, 20-fold induction of defense-related genes like *PR-1, chitinase* and *GST* (Niehl et al., 2006). Interestingly, no correlation occurred between virus titers and defense gene expression in systemic leaves, suggesting that these plant responses are directed primarily against oxidative stress rather than against the invading virus. Furthermore, responses of two potato cultivars (Igor and Nadine) were compared to two *Potato virus Y* (PVY) strains, the aggressive PVY^NTN^ and the mild PVY^N^ (Kogovsek et al., 2010). PVY^NTN^-inoculated leaves displayed chlorotic and/or necrotic ringspot type lesions, while PVY^N^ caused a mild chlorotic ringspot. Potato cv. Igor plants infected by PVY^NTN^ showed a higher expression of antioxidant-encoding genes (*APX, GR* and *GST*) than plants infected with the mild PVY^N^ strain. Interestingly, in PVY-infected cv. Nadine the response was the opposite (Kogovsek et al., 2010), suggesting that host-dependent differential patterns of antioxidant induction could contribute to altered symptom severity in response to different PVY isolates. This is likely also the case during systemic viral infections that result in severe oxidative stress (cell/tissue necrosis), a usual indication of late and failed attempts by the host to induce resistance (Hernández et al., 2016; Künstler et al., 2016). For example, in pea plants susceptible to *Plum pox virus* (PPV), systemic PPV infection produced chlorotic and necrotic lesions, a pronounced oxidative stress indicated by increased protein oxidation, lipid peroxidation, elevated H_2_O_2_ levels and electrolyte leakage in infected leaves (Díaz-Vivancos et al., 2008). Although activities of certain antioxidant enzymes (APX, peroxidase) increased, catalase and GST activities decreased. On the other hand, rice plants systemically infected by *Rice black-streaked dwarf virus* (RBSDV) displayed an induction of *GST23* and the corresponding transcripts, concomitant with oxidative stress (Xu et al., 2013).

In summary, plant GSTs may participate in the establishment of resistance to virus infections, either in the presence or absence of oxidative stress (HR-type necrosis) but could also contribute to the limitation of oxidative stress during virus susceptibility, i.e., in systemic infections. In fact, GSTs, in concert with GSH, could contribute to virus susceptibility in an even more general sense by supporting optimal subcellular conditions for virus replication. It has been shown that the expression of *NbGSTU4* was up-regulated by *Bamboo mosaic virus* (BaMV) in *N. benthamiana*. NbGSTU4 binds to the 3′ untranslated region (UTR) of BaMV positive sense (+) RNA in a GSH-dependent manner and is necessary for efficient viral RNA replication i.e., production of a viral negative sense (–) RNA and then new genomic (+) RNA (Chen et al., 2013). GSH was shown to stimulate *in vivo* BaMV replication and *in vitro* (–) RNA synthesis, while oxidative agents inhibit *in vitro* (–) RNA synthesis (Chen et al., 2013). NbGSTU4 induced by BaMV may provide an antioxidative environment for BaMV RNA replication to eliminate oxidative stress that could be induced by BaMV infection. Therefore, certain plant GSTs may bind viral RNA and deliver GSH to the replication complex thus creating reduced conditions for an efficient viral RNA synthesis.

## Resistance-Inducing Symbiotic Microorganisms and Plant GSTs

Non-pathogenic, symbiotic bacteria and fungi living in the rhizosphere of plants can be highly beneficial to plants attacked by pathogenic microorganisms. These symbiotic microorganisms can produce antimicrobial toxins that are released into the soil and thus restrain pathogens. Furthermore, they are able to activate biochemical defense pathways of plants. This phenomenon is known as induced systemic resistance (ISR) (Pieterse et al., 2014).

The induction of *GST* genes or elevated GST activities has often been observed in plants treated with beneficial bacteria (Hassan et al., 2015; Agisha et al., 2017). Thus, the application of the well-known symbiotic, ISR-inducing rhizobacterium *Pseudomonas fluorescens* to the phyllosphere of an apple scab-susceptible apple (*Malus domestica*) cultivar led to the up-regulation of genes encoding proteins participating in pathogen recognition, signaling and antimicrobial defense such as PR-proteins, thioredoxin-like proteins, heat shock proteins and a GST (Kürkcüoglu et al., 2007). In rice plants, inoculation with *P. fluorescens* led to the accumulation of 23 rice proteins including a GST (Kandasamy et al., 2009). In wheat roots colonized by *P. fluorescens* an antifungal metabolite was identified that suppresses soil-borne root pathogens and activates host defense reactions. In addition, the beneficial bacterium up-regulated the expression of several defense genes encoding PR-10a, the antioxidative monodehydroascorbate reductase enzyme and two GSTs (Maketon et al., 2012). Another important beneficial bacterium, the endophytic *Pseudomonas putida* strongly increased the drought tolerance of chickpea. This beneficial effect was supposedly due to the increased expression of genes involved in biotic stress response (*PR1*), ethylene biosynthesis and ROS scavenging including a *GST* (Tiwari et al., 2016). Colonization of black pepper by *P. putida* led to the induction of several host genes that encoded defense-related proteins such as PR-1, PR-4, catalase, metallothionein, and a GST (Agisha et al., 2017). These transcriptional changes including the induction of *GSTs* may significantly increase plant disease resistance. Indeed, it was observed that the inoculation of wheat roots with *P. fluorescens* markedly suppressed the infection caused by the fungus *Gaeumannomyces graminis* var. *tritici* (Ggt) on the roots. During the early phase of this tripartite interaction, a wheat *GST* gene was induced by Ggt alone while in a later phase of infection the *GST* gene was up-regulated also by *P. fluorescens*. In contrast to *GST*, the expression of two host genes encoding an enolase and a cinnamyl alcohol dehydrogenase did not change significantly during this tripartite interaction (Daval et al., 2011).

Beneficial, symbiotic fungi can not only promote plant growth and nutrient uptake but they are also able to induce key defense reactions in plants including the activation of *GSTs*. Thus, application of the biocontrol agent *Trichoderma harzianum* to cabbage (*B. oleracea* var. *capitata*) plantlets induced resistance against the soil-borne fungal pathogen *R. solani*. The beneficial fungus markedly attenuated the host tissue damage (necrosis) elicited by *R. solani* infection. Concomitantly with the development of resistance the up-regulation of a hydrogen-peroxide inducible *GST* was observed that might contribute to the elimination of cytotoxic reactive metabolites containing an electrophilic moiety (Shibu et al., 2012). Application of *T. harzianum* markedly increased the growth of melon and activated several GSH-related enzymes such as DHAR and GST in melon leaves (Bernal-Vicente et al., 2015). Furthermore, *Trichoderma velutinum* markedly suppressed the infection caused by *R. solani* in common bean and markedly induced the expression of several defense genes including *GSTs* (Mayo et al., 2016).

The endophytic root-colonizing fungus *Piriformospora indica* can markedly promote plant growth and enhance the tolerance of host plants against abiotic and biotic stress. These beneficial effects were attributed to the elevated antioxidative capacity of *P. indica*-inoculated plants due to an activation of GSH-dependent antioxidative pathways (Waller et al., 2005; Harrach et al., 2013). Thus, the significant up-regulation of a tau-class *GST* (*BcGSTU*) was observed in *P. indica*-treated Chinese cabbage roots (Lee et al., 2011; Kao et al., 2016). The overexpression of *BcGSTU* in *A. thaliana* resulted in the stimulation of plant growth and increased resistance against *Alternaria brassicae* infection. This increased resistance against the fungal pathogen was explained by elevated levels of GSH, auxin, SA and jasmonic acid in host tissues. It was supposed that this GSTU enzyme contributed to a balance between growth and defense responses (Kao et al., 2016). Furthermore, the accumulation of two GST proteins was explored by a proteomic study in *A. thaliana* roots inoculated with *P. indica* (Peskan-Berghofer et al., 2004).

In conclusion, the activation of *GSTs* together with other host genes encoding antioxidative and defense enzymes has been often observed during plant-symbiotic microbe interactions that resulted in enhanced resistance against microbial pathogens. However, the exact role of GST enzymes in the mechanism of ISR is still far from elucidated because transgenic plants overexpressing or suppressing the symbiont-inducible *GSTs* have been rarely studied (Kao et al., 2016). GSTs may participate in the detoxification of microbial toxins or in antioxidative reactions.

## Conclusions and Future Perspectives

Since the beginning of plant GST research in 1970 the fundamental questions have remained largely unanswered: what are the physiological roles of GST isoenzymes and which metabolites are the natural, endogenous substrates of GSTs? In particular, what are the exact functions of distinct GSTs in conferring pathogen resistance and/or alleviating oxidative stress in the host? The marked induction of *GST* genes has been often observed in various plant-pathogen interactions, but these observations were rarely followed by functional studies. Thus, the cellular function of most plant GST enzymes in plant-pathogen interactions has remained elusive. Nevertheless, the profile of pathogen-inducible *GSTs* could provide a characteristic signature for a particular plant-pathogen interaction. Obviously, the large number of GST isoenzymes presents a challenge when studying the functions of GSTs in infected plants due to the high likelihood of functional redundancy. The presence of multiple GSTs with overlapping functions and substrate specificities might preclude the observation of phenotypic alteration in knockout mutants (Sappl et al., 2009). Furthermore, in spite of considerable research efforts (Dixon et al., 2009, 2010), only a few endogenous GST substrates have been identified.

We propose a model describing the diverse roles of plant GSTs in the interactions of plant hosts with pathogenic microbes considering four different plant-pathogen interaction types (Table 1): (1) symptomless resistance (including basal resistance to bacteria and symptomless *R* gene-mediated resistance to viruses), (2) HR-associated resistance, (3) limiting susceptibility to systemic spread of pathogens and plant cell/tissue death (during infections by hemibiotrophic/necrotrophic fungi, bacteria, and viruses), (4) promoting susceptibility to biotrophic fungi and viruses (maintaining reduced conditions in infected non-necrotic plant tissues). Certain biochemical and physiological functions of plant GSTs are characteristic of a given plant-pathogen interaction type (e.g., glucosinolate metabolism, detoxification of mycotoxins), while other functions may be common for several or all interaction types, e.g., the control of plant cell death (oxidative stress) by GSTs and regulation of plant GSTs by various hormones and transcription factors. Overall, probably the most important function of GSTs in influencing the outcome of plant-pathogen interactions is the suppression of oxidative stress in infected host tissues (Edwards et al., 2000; Wagner et al., 2002; Gullner and Komives, 2006).

**Table 1:** A model of diverse roles of plant GSTs in four different interaction types between plant hosts and pathogenic microbes.

**Role of plant GSTs**	**Fungal and oomycete infections**	**Bacterial infections**	**Viral infections**
**SYMPTOMLESS RESISTANCE**			
Maintaining resistance/preventing localized cell death (oxidative stress)	Pislewska-Bednarek et al., 2018	Szatmári et al., 2006	Gullner et al., 1995b; Larson et al., 2008; Satoh et al., 2013; Wu et al., 2013a,b
GSTs and auxin	–	Szatmári et al., 2006	–
GSTs and glucosinolate metabolism	Pislewska-Bednarek et al., 2018	–	–
**HR-ASSOCIATED RESISTANCE**			
Maintaining resistance/preventing spread of localized cell death (oxidative stress)	Mauch and Dudler, 1993	Levine et al., 1994; Sarowar et al., 2005	Gullner et al., 1995a,b; Fodor et al., 1997
	Pei et al., 2011; Wang et al., 2012	Kuzniak and Sklodowska, 2004	Király et al., 2012; Widana Gamage et al., 2016
Differential ROS accumulation	Li et al., 2011	Levine et al., 1994; Rajasekhar et al., 1999	Pogány et al., 2004
GST regulation by WRKY TFs	Li et al., 2004, 2006	–	–
GST regulation by PR1	Sarowar et al., 2005	Sarowar et al., 2005	–
GST regulation by SA and ethylene	–	Lieberherr et al., 2003; Blanco et al., 2009	–
**LIMITING SUSCEPTIBILITY**			
Controlling (limiting) spread of cell death (oxidative stress) and pathogens in infected, necrotic plant tissues	Schenk et al., 2000; Dean et al., 2005; Li et al., 2013; Shi et al., 2014; Han et al., 2016; Gong et al., 2018	Mittal and Davis, 1995; Venisse et al., 2001	Kogovsek et al., 2010; Xu et al., 2013
GST regulation by WRKY TFs	Shimono et al., 2007; Shi et al., 2014	Shi et al., 2014	–
GST regulation by SA and ethylene	Shimono et al., 2007; Han et al., 2016	–	–
SA regulation by GST	Gong et al., 2018	–	–
GST catalyzing cinnamic acid-GSH conjugation	Edwards and Dixon, 1991	–	–
GST catalyzing detoxification of mycotoxins	Gardiner et al., 2010; Wahibah et al., 2018	–	–
**PROMOTING SUSCEPTIBILITY**			
Maintaining reduced conditions (preventing cell death) in infected, non-necrotic plant tissues	El-Zahaby et al., 1995; Harrach et al., 2008; Hernández et al., 2009	–	Love et al., 2005; Casado-Vela et al., 2006
			Niehl et al., 2006; García-Ibarra et al., 2011
			Skopelitou et al., 2015; Pavan Kumar et al., 2017

In the case of several plant-pathogen interactions, transgenic plants overexpressing or silenced for individual *GSTs* have been useful tools to study resistance mechanisms. In addition, the comparison of *GST* up-regulations between compatible and incompatible plant-pathogen interactions has also proved that GSTs can contribute to disease resistance, however, most of the underlying molecular mechanisms are still not completely known. For example, we need to gain more information on the regulation of *GST* expression during incompatible plant-pathogen interactions. In addition, further studies are needed to elucidate the regulatory elements in the 5′ flanking promoter regions of *GST* genes that are responsive to various infections. Once these cis-acting regulatory elements are identified, the transcription factor proteins required for transcriptional activation can be also determined. The tight metabolic links between GSTs and plant defense hormones, particularly SA, should be more deeply understood. The future characterization of the fascinating, large and diverse GST family will fill in many gaps in our knowledge on plant signaling processes, defense responses and disease resistance.

## Author Contributions

GG and PS conceived the idea of the manuscript, wrote the introduction and the conclusions as well as prepared the figures. GG wrote the plant-fungus section. TK discussed the plant-bacterium interactions, while LK prepared the plant-virus section.

### Conflict of Interest Statement

The authors declare that the research was conducted in the absence of any commercial or financial relationships that could be construed as a potential conflict of interest.
